# Challenges associated with the implementation of institutional quarantine and isolation strategies during major multicountry viral outbreaks in Africa (2000–2023): a scoping review

**DOI:** 10.1186/s41256-024-00385-8

**Published:** 2024-10-18

**Authors:** Jimoh Amzat, Ebunoluwa Oduwole, Saheed Akinmayowa Lawal, Olusola Aluko-Arowolo, Rotimi Afolabi, Isaac Akinkunmi Adedeji, Ige Angela Temisan, Ayoyinka Oludiran, Kafayat Aminu, Afeez Abolarinwa Salami, Kehinde Kazeem Kanmodi

**Affiliations:** 1 Department of Sociology, Usmanu Danfodiyo University, Sokoto, Nigeria; 2 Department of Sociology, University of Johannesburg, Johannesburg, South Africa; 3 School of Health and Life Sciences, Teesside University, Middlesbrough, UK; 4 Department of Philosophy, Olabisi Onabanjo University, Ago-Iwoye, Ogun State Nigeria; 5 Department of Sociology, Olabisi Onabanjo University, Ago-Iwoye, Ogun State Nigeria; 6 Department of Epidemiology and Medical Statistics, College of Medicine, University of Ibadan, Ibadan, Nigeria; 7 Department of Science and Technology Education, University of Ibadan, Ibadan, Nigeria; 8 Center for Child and Adolescent Mental Health, University College Hospital, Ibadan, Nigeria; 9 Faculty of Dentistry, University of Puthisastra, Phnom Penh, Cambodia; 10 Department of Public Health Dentistry, Manipal Academy of Higher Education, Manipal, India; 11 Department of Oral and Maxillofacial Surgery, University College Hospital, Ibadan, Nigeria; 12 Department of Preventive and Community Dentistry, University of Rwanda, Kigali, Rwanda

## Abstract

**Background:**

Quarantine and isolation (Q&I) are interrelated but not mutually exclusive public health practices for disease control, which may face public resistance in the context of health emergencies due to associated challenges. Hence, it is often tough for most countries to implement Q&I even in the context of health emergencies. Therefore, this scoping review examines the challenges associated with the implementation of institutional Q&I strategies during major multicountry viral outbreaks (Ebola, Lassa and COVID-19) in Africa between 2000 and 2023.

**Methods:**

This scoping review was designed based on Arksey and O’Malley’s guidelines. A systematic literature search, using nine online research databases, was conducted with the aid of relevant search terms, Boolean operators and truncations. All articles obtained from the literature search were electronically imported into Rayyan web application for deduplication based on specific inclusion and exclusion criteria. From the included literature, relevant data were charted, summarized, collated, and presented.

**Results:**

This review included 24 of the 787 retrieved articles. Sixteen of the 24 selected articles investigated issues related to COVID-19 prevention and control in Africa. Two assessed precautionary practices for Lassa fever, while five were on Ebola virus disease. However, one article explored knowledge, preventive practices, and general isolation precautions. The review identified various challenges that hindered the implementation of successful Q&I practices during viral infection outbreaks in Africa. Essential healthcare infrastructure, equipment (medical supplies including personal protective equipment and testing kits) and facilities that are essential for Q&I were deficient. Q&I implementation was often threatened by low human resource capacity and inefficiencies in the healthcare system which portray Africa as unprepared to handle complex public health crises.

**Conclusions:**

This review shows that Q&I implementation in Africa is often threatened by low human resource capacity and inefficiencies in the healthcare system and also portrays Africa as unprepared to handle complex public health crises. Hence, Q&I for major multicountry outbreaks in Africa is very challenging. Therefore, continuous efforts to address these identified challenges are crucial to enhancing health emergency preparedness in Africa.

**Supplementary Information:**

The online version contains supplementary material available at 10.1186/s41256-024-00385-8.

## Introduction

Quarantine and isolation (Q&I) have become major control measures in the face of infectious diseases of global significance. Over the last ten years, Q&I have impacted global health space because of their relevance in managing the spread of infectious diseases such as Zika, monkeypox (Mpox), Ebola and coronavirus diseases [1, 2]. Q&I are public health measures or practice designed to protect the public by preventing exposure to people who have, or may have an infectious disease. Quarantine is recommended when a person has been exposed to a highly contagious disease, especially when a person returns from an endemic area or has had contact with an infected person [3]. The practice separates an exposed person at risk, i.e., with a probability of developing the symptom of an infectious disease, from the community. Isolation, on the other hand, is the practice of separating sick or symptomatic persons with a contagious disease from people who are not sick. This includes all measures to exclude asymptomatic (i.e., quarantine) or symptomatic (i.e., isolation) persons returning from regions already affected by an infectious disease from social mingling [4].

Q&I are historical practices that date back to the Mosaic Law described in Leviticus’s book, written around the 7th century. Early Islamic history has also indicated Q&I were practiced at various times [5]. Q&I represent interrelated, but not mutually exclusive, public health practices for disease control, which are poorly understood as disease control strategies. Fundamentally, human mobility is mostly responsible for the spread of infectious agents, especially when outbreaks occur. Hence, the implementation of Q&I and other non-pharmaceutical interventions including lockdown, face-masking, and social distancing, are core measures for preventing the spread of infectious diseases. The Coronavirus Disease 2019 (COVID-19) was a typical pandemic that led to global lockdown by disrupting human mobility. While most health authorities focused on public health measures including Q&I, citizens complained of consequent socioeconomic and political challenges, resulting in compliance problems [6].

Institutional Q&I are mostly provided by the state, with support from non-state actors, through formalized facilities to hospitalize persons of interest for care within a specified period. In this scenario, the person might not be permitted to self-quarantine at home. This mostly happens at the beginning of any outbreak for effective containment of further spread. Most places resist Q&I strategies during health emergencies, such as Ebola and COVID-19 [5]. Citizens are transferred to isolation centers, while infected but asymptomatic persons may practice self-isolation and self-medication [6]. In some cases, infected persons have fled from isolation centers [7]. Beyond the implementation or enforcement by the authority, Q&I are acts of responsibility and protection for others and other community members.

However, Q&I strategies raise ethical issues, as there are nuances of abuse and inequality. Q&I deliberately impair the rights to movement and association to ensure the safety of others in the community. The ethical debate is about personal liberties and the public good. The outbreak of infectious disease presents ethical dilemmas when medical protocols involving Q&I are sacrosanct [4]. The exposed or infected person poses a risk to others in society. Other challenges include concerns about the health system’s weakness, and observed inequalities in the quality epidemiology of these disease outbreaks, requiring that health system preparedness mechanisms should be set up for future outbreaks. Hence, conducting a scoping review on the challenges associated with implementing Q&I will allow for a comprehensive understanding of the diverse and complex issues encountered during enforcement, including logistical, ethical, and social issues. By mapping existing literature, future Q&I measures could be better planned and more effective. Ultimately, this is a novel scoping review designed to provide valuable insights concerning informed, equitable, and efficient Q&I implementation during health emergencies. As a result, this scoping review aims to examine the challenges associated with implementing institutional Q&I strategies during major multicountry viral disease outbreaks in Africa, following Arksey and O’Malley’s [8] guidelines. The viral disease outbreaks of interest include Ebola, Lassa and COVID-19 infections which have been major multicountry outbreaks in the last two decades (2000–2023), especially in Africa.

## Methods

### Literature identification

The research question for this scoping review was “What is the empirical evidence on the challenges associated with the implementation of institutional Q&I strategies during major multicountry viral outbreaks in Africa?”. To retrieve literature relevant to the research question, on July 24th, 2023, a systematic literature search was conducted with the aid of relevant search terms, Boolean operators (“AND” and “OR”), and truncations (“*” or “#”) in nine online research databases (APA PsycArticles, APA PsycInfo, PubMed, SCOPUS, Psychology and Behavioral Sciences Collection, Allied and Complementary Medicine Database (AMED), CINAHL Ultimate, Dentistry and Oral Sciences Source, and SPORTDiscus with Full Text). The search terms were obtained from the Thesaurus and Medical Subject Headings (MeSH) dictionary (Tables S1 to S3; Supplementary file).

### Literature selection

All articles obtained from literature search were electronically imported into the Rayyan web application to remove all duplicate records [9]. After this, the deduplicated records were subjected to a two-stage screening process, using a set of inclusion and exclusion criteria, to select the literature that addresses the research question. The first stage involved prima facie evaluation of deduplicated literature through screening of their titles and abstracts. At this stage, non-relevant literature was excluded. The remaining non-excluded literature was thereafter subjected to the second-stage screening process where their full texts were thoroughly read and evaluated for eligibility based on the scoping review’s criteria (Table S4; Supplementary file). The two-stage screening process was carried out by two independent reviewers. During each stage of the screening process, all conflicts in inclusion/exclusion that arose were resolved through brainstorming, critical discussions, and a joint consensus by the reviewers involved.

To be included into this review, the screened literature must be (1) an original research article with accessible full text; (2) published in English and in a peer-reviewed journal; (3) published in the year 2000 or upwards; and (4) report empirical data on the challenges associated with the implementation of institutional Q&I strategies during major multicountry viral outbreaks in Africa. However, the screened literature was excluded if it (1) was grey literature (e.g., book chapters, books, technical reports, etc.); (2) was published in non-English language; (3) did not present empirical data; (4) presented empirical data during major multicountry non-viral outbreaks in Africa; (5) presented empirical data in non-African populations; and (6) published before the year 2000.

The reference lists of the included articles were screened to identify and incorporate any other eligible articles that were not identified through the utilized electronic databases in the scoping review.

### Data charting, collation and summarization

Relevant data were charted, using a customized data extraction sheet, from the included articles. These data include the names of authors, year of publication, study location, study design, study objectives, size of the study sample/population, study population/sample attributes, study tools, empirical findings, and conclusions. The charted data were then collated, summarized, and presented in texts and Tables [10, 11]. The data collation, summarization, and presentation were done using the inductive thematic analysis framework proposed by Braun and Clarke [12, 13]. In this analytic approach, the charted data were first grouped into nodes, after which they were merged into subthemes and themes.

## Results

### Search results and characteristics of included articles

**Fig. 1:**
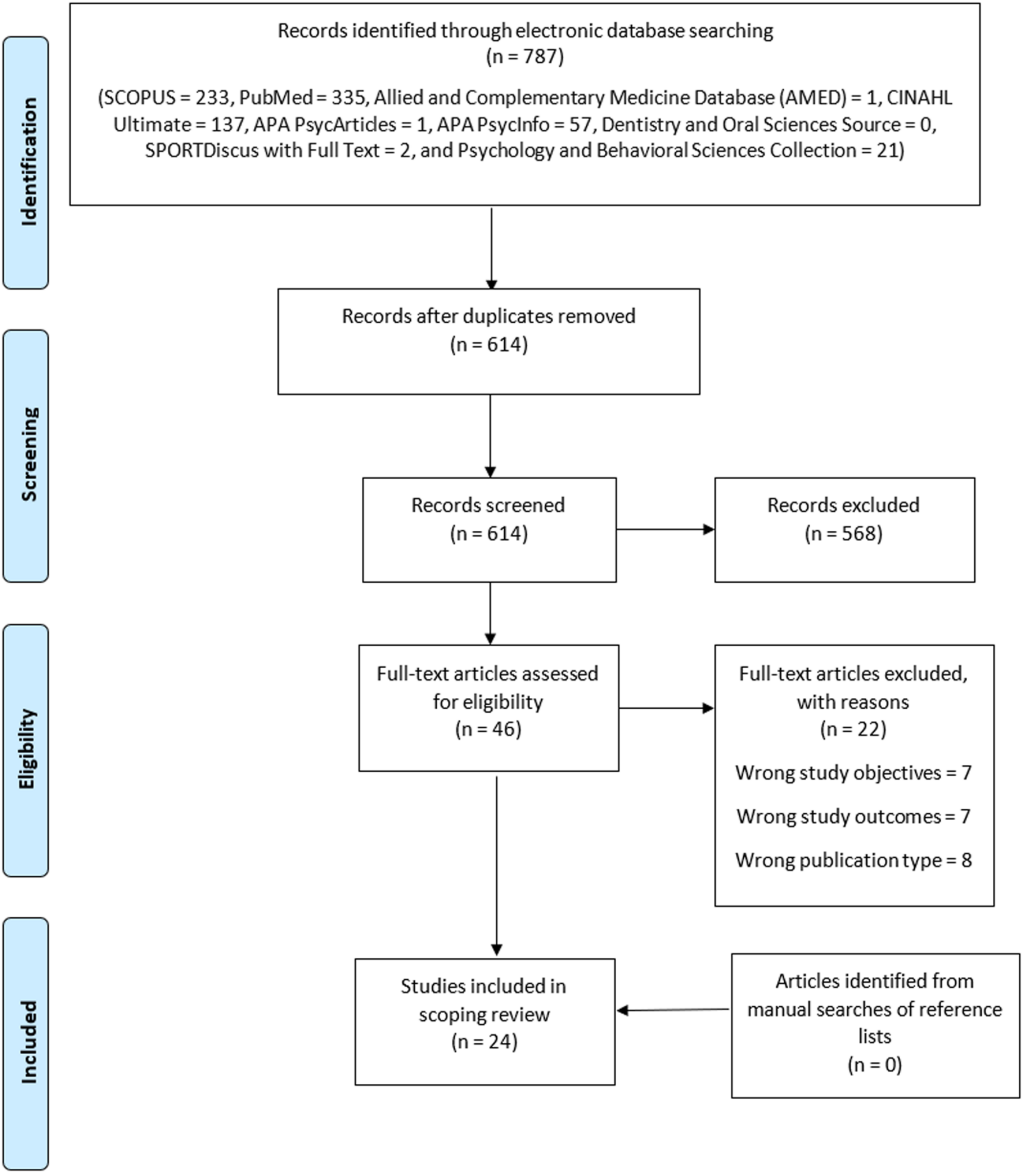
PRISMA flow chart

The literature search yielded a total of 787 articles. After the removal of duplicates, 614 single entries were screened for eligibility. Out of the 614 articles, only 24 were found relevant and included in this scoping review after a two-stage screening process (see Table 1). Also, the reference lists of these 24 articles were manually searched to identify any other relevant articles, but none was found. Finally, this scoping review included a total of 24 articles (see Fig. 1).

Sixteen of the 24 selected articles investigated issues related to COVID-19 prevention and control in Africa [14–28]. Two assessed precautionary practices for Lassa fever [29, 30], while five were on Ebola virus disease control [31–35]. However, one article explored knowledge, preventive practices, and isolation precautions broadly, the authors’ focus was not on a particular infection.

**Table 1:** Publications reviewed on quarantine and isolation implementation in Africa

S/N	References	Study location	Objective(s)	Infection investigated	Study design	Study population	Sample size	Challenges with institutional Q&I	Conclusion
01	Ndoungué et al. [36]	Cameroon	Ascertain the current level of existing International Health Regulations (IHR) core capacities of designated airports, ports and ground crossings in Cameroon; Identify critical gaps for capacity building for prevention, early warning and response to public health threats	COVID-19	Cross-sectional study	Representatives of various institutions working in points of entry	35 representatives from 5 points of entry	Insufficient training on Q&I/ infection prevention and control (IPC) Poor knowledge of IPC Non-compliance to recommended IPC guidelines Shortage of health infrastructure	All facilities should be enhanced as none of those assessed met international health regulation standards and need significant s requirements.
02	Fawole et al. [18]	Democratic Republic of Congo, Nigeria, Senegal, and Uganda	Describes the strengths, weaknesses and lessons learnt from the COVID-19 surveillance strategies	COVID-19	Mixed-methods observational study	Policymakers and members of the national and regional emergency operations center for COVID-19 response; policymakers, epidemic focal persons, and health managers.	120	Low funding Insufficient screening capacity Shortage of health infrastructure	Decentralizing surveillance and private sector involvement will support response activities and ensure proper regulation and quality assurance.
03	Desie et al. [17]	Ethiopia	Examine the coping strategies used by returnees who were in mandatory quarantine	COVID-19	Center-based cross-sectional study	Migrant returnees	405	Negative experiences with the Q&I enforcement	Psychosocial reintegration efforts need to focus on enhancing returnees’ capacity to use adaptive coping strategies
04	GebreEyesus et al. [19]	Ethiopia	Healthcare providers’ preparedness and healthcare protection against the third wave of COVID-19	COVID-19	Institutional-based cross-sectional study	Health care providers	326	Poor knowledge of procedures for assessing patients under investigation Insufficient training on Q&I/IPC Poor knowledge of IPC Inadequate PPE	Government and other stakeholders should design interventions to increase healthcare providers’ preparedness.
05	Habtamu et al. [20]	Ethiopia	Investigate the prevalence of psychological distress and associated factors among migrant returnees who were in quarantine during the time of COVID-19.	COVID-19	Institution-based cross-sectional study	Migrant returnees	405	Negative experiences with the Q&I enforcement	Returnees in quarantine will benefit from screening and integration of mental health support with other services. The burden of psychological distress can be reduced through efficient referral.
06	Misgana et al. [24]	Ethiopia	Assess the psychological burden of COVID-19 on the people in quarantine and isolation centers; To identify associated factors for early and effective psychosocial intervention during the pandemic and beyond	COVID-19	Cross-sectional study	Suspected cases of COVID-19 in isolation/quarantine centers.	392 patients	Negative experiences with the Q&I enforcement	The deleterious effects of COVID-19 on mental health were more pronounced in some population subgroups.
07	Olani et al. [27]	Ethiopia	Explore quarantine experience of people in Southern Nations Nationalities and Peoples’ Region	COVID-19	Exploratory qualitative research design	Quarantined people	29	Negative experiences with the Q&I enforcement	It is important to appraise the psychological, physiological, social, and economic impacts of quarantine on the individuals
08	Adokiya and Awoonor-Williams [31]	Ghana	Assess the EVD surveillance and response system	Ebola virus disease (EVD)	Observational study	Health workers	47	Insufficient training on Q&I/IPC Poor knowledge of IPC Insufficient screening capacity	EVD surveillance and response preparedness were insufficient; surveillance strengthening is imperative
09	Asare et al. [15]	Ghana	Identify the challenges and opportunities associated with COVID-19 contact tracing	COVID-19	Exploratory qualitative design	Contact tracers	39	Low staff strengths Scarce trained personnel Poor staff welfare and protection Inadequate PPE	Health authorities should address identified challenges and make good use of the recommended opportunities
10	Awoonor-Williams et al. [32]	Ghana	Assess the challenges of screening travellers for EVD at border entry in northern Ghana.	Ebola virus disease	Observational study	Port health officers and district directors of health Involved in EVD screening	12 = 7 port health officers and 5 district directors of health)	Low staff strengths Scarce trained personnel Insufficient training on Q&I/IPC Poor knowledge of IPC Shortage of health infrastructure	Screening for Ebola remains sub-optimal at the entry points in northern Ghana due to several factors
11	Delamou et al. [16]	Guinea	Assess levels of health facility preparedness and response to the COVID-19 pandemic.	COVID-19	Cross-sectional Study	Managers and healthcare workers in public and private health facilities/services	197 managers and 1020 HCWs	Inadequate PPE Insufficient screening capacity Shortage of health infrastructure	Domestic funding and better governance should be increased to improve preparedness for future outbreaks.
12	Oji et al. [33]	Liberia	Present the result of IPC capacity-building strategies including IPC training and mentorship	Ebola virus disease	Intervention study	Health care workers	180	Non-compliance to recommended IPC guidelines	Building the capacity of healthcare workers for IPC is essential for safe and quality service delivery.
13	Pellecchia et al. [35]	Liberia	Assess Liberian community perspectives on State-imposed Ebola public health and outbreak containment measures implemented in 2014 and 2015.	Ebola virus disease	Qualitative design	Community members	462	Inadequate essential needs for the patients	Efforts in the direction of awareness and community involvement are potential better strategies to control EVD epidemic.
14	Adebimpe and Ibirongbe [37]	Nigeria	Explore the knowledge and preventive practices on isolation precaution (IP) and quarantine among healthcare workers	Not specified; however, the study was conducted to evaluate the response to a recent Ebola and Lassa fever outbreak	Explorative cross-sectional study	Health care workers	800	Insufficient training on Q&I/IPC Poor knowledge of IPC	Building the capacity of all health workers on isolation and quarantine measures would reduce infection transmission in hospital settings
15	Aidonojie et al. [14]	Nigeria	Examining legal constraints and ethical issues concerning the care of COVID-19 patients by private healthcare	COVID-19	Hybrid research design (doctrinal and empirical research approaches)	Community members	314	Shortage of health infrastructure	There is a need to whittle down the laws and regulations limiting private health providers from caring for COVID-19 patients
16	Ijarotimi et al. [29]	Nigeria	Assess the knowledge of Lassa fever among health care workers, availability of IPC measures and their use during Lassa fever outbreak	Lassa fever	Descriptive cross-sectional study	Healthcare workers and health facilities	190 health personnel; 59 health facilities	Insufficient training on Q&I/IPC Poor knowledge of IPC Non-compliance to recommended IPC guidelines Inadequate PPE Shortage of health infrastructure	IPC committee and training should be mandatory in every location
17	Joy Okwor et al. [22]	Nigeria	Describe the state of IPC preparedness within health facilities in Nigeria during the early phase of coronavirus disease	COVID-19	Cross-sectional survey	Health facilities; managers of health facilities	461	Inadequate Outbreak Response Preparation Lack of personal protective equipment (PPE) Insufficient training on Q&I/IPC Poor knowledge of IPC Poor staff welfare and protection	Creation of conducive IPC structures will minimize COVID-19 transmission risks in healthcare settings.
18	Obionu et al. [30]	Nigeria	Evaluate IPC practices during Lassa fever outbreak.	Lassa fever	Cross-sectional survey	Lassa fever treatment centers; IPC focal persons	2 Lassa fever treatment centers	Low staff strengths Scarce trained personnel Insufficient training on Q&I/IPC Poor knowledge of IPC Low funding Non-compliance to recommended IPC guidelines	Compliance with IPC guidelines should be mandated in all treatment centers.
19	Ogoina et al. [26]	Nigeria	Assess the readiness of hospitals in Nigeria to respond to the COVID-19 outbreak	COVID-19	Descriptive cross-sectional study	Public hospitals (COVID-19) designated	20 hospitals (17 tertiary and 3 secondary)	Insufficient training on Q&I/IPC Poor knowledge of IPC Inadequate PPE Insufficient screening capacity Shortage of health infrastructure	Efforts to strengthen hospital preparedness should prioritize challenges related to surge capacity, critical care for COVID-19 patients, and staff welfare and protection.
20	Sanni et al. [28]	Nigeria	Evaluates aspects of the school health program (SHP) in some selected Nigerian schools that might relate to the pandemic control during school re-opening.	COVID-19	Cross-sectional survey design	Primary schools	146 Primary schools	Infrastructural deficits and gaps in policy to practice Low funding Inadequate PPE Shortage of health infrastructure	Barriers to safe school re-opening Include infrastructural deficits and gaps in policy to practice. It is essential to strengthen the local SHP to build resilience against future epidemics.
21	Pedi et al. [34]	Sierra Leone	Explain the rationale for a standardized approach, (b) describe the methodology used to develop the resulting SOPs, and (c) discuss the implications of the SOPs for future outbreak responses	Ebola virus disease	User-centered design	Individuals active in social mobilization and community engagement	250	Negative social factors including mistrust	Experience points to the need for a set of global principles and standards for meaningful community engagement that is adaptable as a high-priority response component at the outset of future health and humanitarian crises.
22	Idrees & Bashir [21]	Sudan	Explore the psychological problems in terms of anxiety and depression among healthcare workers in COVID-19 treatment centers	COVID-19	Cross-sectional study	Healthcare workers at isolation centers	133	Negative experiences with the Q&I enforcement	COVID-19 pandemic impacts the mental health of frontline workers.
23	Kharroubi et al. [23]	Tunisia	Describe the mental health status of adults under mandatory institutional quarantine imposed during the COVID-19 pandemic, and determine factors influencing the occurrence of psychiatric symptoms	COVID-19	Cross-sectional phone survey	Adults under compulsory institutional quarantine	506	Inadequate Outbreak Response Preparation Negative experiences with the Q&I enforcement	Psychological interventions should thus be an integral part of the COVID-19 control strategy
24	Ndejjo et al. [25]	Uganda	Experiences of individuals who underwent institutional quarantine	COVID-19	Qualitative description study	Individuals who had been quarantined in institutional facilities	20	Negative experiences with the Q&I enforcement	Planning, management, and implementation of the quarantine process will determine people’s experience of quarantine

Seven studies were conducted in Nigeria [14, 22, 26, 28–30, 37], 5 in Ethiopia [17, 19, 20, 24, 27], 4 in Ghana [15, 31, 32], 2 in Liberia [33, 35], and 1 each in Sudan [21], Guinea [16], Sierra Leone [34], Uganda [25], Cameroon [36], and Tunisia [23]. One was a multicountry study conducted in 4 different locations—the Democratic Republic of Congo, Nigeria, Senegal and Uganda [18].

Units of analysis varied amongst the reviewed articles. The majority of the sampled individuals included healthcare personnel, persons who experienced or were in quarantine, migrants, community members, policymakers, epidemic focal persons, and some aimed at health managers (see Table 1). Furthermore, some articles included samples from primary schools, hospitals, community mobilizers, and contact tracers. In total, 5882 persons, 81 health facilities, and 146 primary schools were respondents. Only one of the 23 articles introduced intervention in their research [33].

### Challenges with Q&I implementation during viral infection outbreaks in Africa

#### Inadequate outbreak response preparation

Several African countries exhibited unpreparedness during the COVID-19 outbreak. For instance, in Nigeria, the overall level of preparedness among healthcare institutions was inconsistent [22, 26]. Many hospitals lacked isolation units until after the virus was confirmed in the country, with only 45% of hospitals establishing such facilities [26]. In a sample of 20 hospitals, only 15% were highly prepared, 75% were moderately prepared, and 10% were classified as not ready based on World Health Organization (WHO) standards [26]. In Ethiopia, over one-third of healthcare workers rated their facility’s preparedness as poor, citing a lack of isolation and triage protocols [19]. Similarly, preparedness at points of entry in Cameroon was inadequate, particularly in areas such as communication, resource evaluation, and sanitary inspection [36]. The lack of comprehensive plans for responding to outbreaks at both the community and state levels contributed to ineffective Q&I implementation.

#### Human resource challenges

A shortage of trained personnel hindered the management of viral outbreaks in several African countries. In Nigeria, for example, there was a limited availability of infectious disease specialists, with anesthesiologists being particularly scarce [26]. In Ghana and other countries such as Senegal, Uganda, and the Democratic Republic of Congo (DRC), the scarcity of trained personnel affected contact tracing and other public health activities essential for managing Ebola Virus Disease (EVD) and COVID-19 [15, 18, 32]. In Nigeria, nearly half of health workers had not been trained in general infection prevention and control (IPC) measures [37]. Similarly, in Cameroon, none of the healthcare workers had been trained in surveillance activities, and less than half of healthcare workers in Guinea received any formal training in COVID-19 prevention and management [16]. In Ethiopia, while many healthcare workers had undergone training, gaps in knowledge persisted, particularly in isolation techniques and procedures for reporting suspected COVID-19 cases [19].

The knowledge and application of IPC measures were found to be suboptimal across multiple African countries. In Nigeria, for instance, the knowledge of IPC practices among healthcare workers was poor, with 82% of personnel being unaware of proper isolation precautions, and only 7.6% understanding when personal protective equipment (PPE) should be used [37]. Similarly, in Ethiopia, nearly half of the healthcare personnel lacked skills in isolation techniques and methods for reporting suspected COVID-19 cases [19]. The overall understanding of EVD screening protocols and IPC measures was also low among health workers in Ghana, affecting their ability to screen migrant returnees effectively [32].

The welfare and protection of healthcare workers were inadequate in several African countries during viral outbreaks. For example, in Nigeria, many hospitals lacked provisions for staff accommodation, feeding, and life insurance for personnel managing COVID-19 patients [26]. In Ghana, contact tracers complained about poor remuneration and a lack of insurance coverage [15]. The shortage of PPE further compounded the challenges. In Nigeria, Ethiopia, and Guinea, many hospitals lacked sufficient PPE for their personnel [16, 19, 26]. In Guinea, 70% of healthcare workers had not received PPE for three months, raising concerns about their safety while managing COVID-19 patients [16]. Similarly, healthcare workers in Ghana expressed anxiety about their safety due to inadequate PPE when screening for EVD [32].

Noncompliance with IPC guidelines was another major issue affecting Q&I implementation. In Nigeria, some health workers failed to follow basic precautions such as handwashing, wearing facemasks, or using PPE when attending to patients [30]. Compliance with IPC measures was found to be better at designated Lassa fever treatment centers than at non-designated centers [22]. In Liberia, an intervention study revealed low baseline compliance with IPC practices, though some improvements were observed post-intervention [33]. In some countries, such as Guinea, hospitals had yet to receive necessary guidance documents for COVID-19 prevention, sample collection, and patient management, further impeding compliance with IPC measures [16].

#### Healthcare infrastructure shortages

The lack of adequate healthcare infrastructure was a persistent challenge in many African countries. In Nigeria, Lassa fever treatment centers were found to lack basic amenities such as perimeter fences, hand hygiene facilities, and separate toilets for infected patients [30]. In Ghana, health personnel relied solely on thermometers for screening EVD cases due to a lack of laboratory testing capacity [31]. The absence of dedicated spaces for Q&I was a significant problem in Guinea, Nigeria, Cameroon, and Ghana. In Guinea, 74% of health facilities lacked dedicated spaces for isolating confirmed COVID-19 cases [16]. Similarly, in Nigeria, 83.5% of healthcare institutions were found to be suboptimal for COVID-19 patient care due to a lack of isolation facilities, bed space, and oxygen support [14].

Screening for viral infections was hindered by a lack of resources and equipment in several African countries. In Guinea, 93% of hospitals had no equipment to screen for COVID-19 [16]. Similarly, in Nigeria, a significant number of hospitals lacked the resources to test for COVID-19 [22, 26]. The lack of laboratory support was also a problem in Ghana, where health personnel could not test suspected EVD cases, relying solely on thermometers for screening [31]. Testing shortages were also reported in the DRC, where insufficient test kits affected outbreak control efforts [18]. The inability to detect suspected cases of infection in a timely manner led to underreporting and a delay in referring confirmed cases to appropriate care centers in countries like Senegal and Uganda [18].

The shortage of health infrastructure is also a function of funding constraints. Hence, limited funding was a critical issue affecting Q&I implementation in Africa. The renovation of Lassa fever treatment centers in Nigeria, for example, was stalled due to a lack of financial resources [30]. Similarly, inadequate funding forced countries such as Nigeria to shift from institutional Q&I to self-quarantine and self-isolation measures during the COVID-19 pandemic. In the DRC, insufficient funding affected outbreak control efforts, while in Uganda, overreliance on donor funding disrupted surveillance and infection control measures [18]. The lack of sustainable funding models for public health infrastructure and personnel support remains a significant barrier to effective outbreak management in Africa.

#### Social factors affecting Q&I implementation

Social and cultural factors such as stigma and misinformation significantly hampered the effectiveness of Q&I in African nations. In Nigeria and Senegal, for instance, the stigma associated with being infected led to underreporting of cases, making it difficult to track and isolate the spread of infections. Nigeria’s vast landmass, negative perceptions of COVID-19, and the inaccessibility of certain conflict zones in northern regions compounded the challenges of effective outbreak control [18]. Similarly, in Sierra Leone, health personnel struggled to reach vulnerable populations in remote areas, which limited the country’s ability to contain the virus effectively [34].

Another significant issue was poor self-quarantine practices, where individuals exposed to the virus continued to interact with others to meet daily needs, potentially spreading the virus further [15, 18]. This was exacerbated by harassment of contact tracers, with quarantined individuals demanding their test results and food [15]. In countries like Ghana, porous borders, uncooperative travelers, stockouts of essential materials, and language barriers further complicated containment efforts during the EVD outbreak [32]. Political discourse around disease outbreaks was also politicized, further hindering cooperation between health workers and the public [15].

In Liberia, state-enforced quarantines heightened stigmatization and mistrust within communities. This led to panic, fear, and the disenfranchisement of vulnerable groups. The practice of mandatory cremation during the EVD outbreak, as well as the enforcement of quarantine measures, were perceived as degrading, resulting in secret burials and further distrust in the system [35]. Essential supplies, such as food and water, were often rationed, leading to non-compliance with quarantine rules in Liberia [35]. Sierra Leone faced additional challenges, including poor social mobilization, weak community engagement, and a lack of two-way communication between health officials and local communities. This lack of dialogue resulted in ineffective responses and a general distrust in health interventions [34].

#### Negative experiences with Q&I enforcement

Enforcement of Q&I measures often resulted in negative experiences for those affected. In many cases, quarantine conditions were substandard, with individuals facing boredom, poor hygiene, unhealthy meals, and limited access to drinking water. These poor conditions, combined with preferential treatment for certain individuals and the high cost of quarantine, led to widespread dissatisfaction [25]. Communication gaps regarding quarantine protocols, such as preparation, length of stay, and the collection of COVID-19 test results, created confusion and anxiety for those in quarantine [25]. In Uganda, quarantined individuals expressed concerns about stigma and the fear of being attacked post-discharge. A significant portion (43.7%) feared discrimination upon their release from isolation [17]. In Ethiopia, many quarantined individuals (85.2%) struggled with financial insecurity during and after their stay in quarantine, with 64% lacking any plans for life post-quarantine. This experience was exacerbated by the inability to engage in normal social interactions [20]. Quarantine hesitancy, contact denial, and mistreatment by law enforcement were additional negative experiences reported in Uganda, with some individuals being mistakenly quarantined due to errors in identity [27].

Healthcare personnel also faced significant challenges. In Sudan, nearly half of healthcare workers (48%) were concerned about contracting the virus, and many reported experiencing anxiety and an increased workload due to the pandemic [21]. Similarly, some quarantined patients in Uganda experienced heightened anxiety about the possibility of infection during their isolation [25].

#### Psychosocial burden of Q&I

The psychosocial burden of Q&I on individuals and healthcare workers was another major challenge. In Uganda, psychological distress was prevalent, primarily driven by fear of infection. Quarantined individuals reported sleep disturbances, poor appetite, weight loss, and social isolation due to stigma and loneliness. Many also suffered from economic losses due to the inability to work during their quarantine period [27]. In Ethiopia, common mental disorders were widespread among quarantined individuals, with symptoms of depression (55%), anxiety (48.9%), and stress (35.6%) being reported among migrant returnees [17]. The psychosocial burden was further compounded by social disruptions, with individuals fearing stigma and grappling with economic difficulties [17]. The psychosocial impact of COVID-19 isolation on patients was influenced by several factors. These included individual characteristics such as gender, pre-existing chronic illnesses, poor awareness of the outbreak, and substance use [24]. In Tunisia, individuals in institutional quarantine experienced clinical insomnia (19.2%), anxiety (15.4%), and depression (37.4%). Students, young adults, and those who feared contracting the virus while in quarantine were more susceptible to anxiety and depression. Individuals who stayed in containment zones throughout their quarantine were more likely to suffer from clinical insomnia [23].

In Ethiopia, a significant correlation was found between depressive symptoms and factors such as fear of infection, inadequate information about quarantine, and concerns about discrimination post-quarantine. Gender (female) and the experience of COVID-19-like symptoms during quarantine were also associated with anxiety symptoms [20]. Similarly, in Sudan, healthcare personnel experienced high levels of anxiety and depression, with women being more likely to suffer from these mental health challenges [21]. Various coping strategies were adopted by individuals during Q&I. These strategies included emotion-focused, problem-focused, and avoidance-focused approaches. Some individuals maintained regular contact with loved ones, engaged in daily routines, exercised, or stayed busy with work or studies to cope with the psychological and social stress of quarantine [25]. In Uganda, personal and social support networks played a critical role in helping individuals cope with the challenges of quarantine. Spiritual strengthening, problem-solving, and peer support were also effective coping mechanisms [27].

## Discussion

This review examines implementation of Q&I measures during viral infection outbreaks in Africa, synthesizing findings from 24 studies. It focuses on the numerous public health challenges experienced on the continent, such as the Ebola virus, Lassa fever, Zika virus, Mpox, and the COVID-19 pandemic. Since 2001, Africa has witnessed over 1,800 public health events, demonstrating the continent’s vulnerability to infectious diseases [35, 36]. In the context of infectious disease outbreaks, international organizations such as the Centers for Disease Control and Prevention (CDC) and the WHO have issued IPC guidelines. These recommendations include measures like hand and respiratory hygiene, the use of PPE, environmental sanitation, waste management, disinfection, and sterilization procedures, as well as adherence to precautions when implementing Q&I measures [37–39]. Ensuring compliance with these guidelines through hospital audits is critical for curbing disease transmission and ensuring the safety of healthcare personnel and the public.

Q&I are non-pharmaceutical public health interventions aimed at reducing close contact between individuals to prevent the spread of infections. Quarantine typically involves individuals who may have been exposed to a disease but are not yet symptomatic, while isolation applies to individuals with a confirmed infection [40, 41]. The goal of both interventions is to limit community transmission of infections, which is in the public’s best interest [40]. The review found that healthcare infrastructure in Africa is often inadequate to support effective Q&I. Many facilities lack dedicated spaces for isolating suspected and confirmed cases, while resources such as medical supplies, hygiene items, and PPE are insufficient to meet international IPC standards. This shortage of infrastructure and supplies increases the risk of nosocomial infections—those contracted in hospitals—especially during major outbreaks like COVID-19, Ebola virus disease, and Lassa fever [42–47]. Several studies in the review confirmed that these infrastructural challenges are common across Africa, contributing to the continent’s high vulnerability to infectious diseases [41–45].

Human resource limitations also pose a significant barrier to effective Q&I implementation in Africa. Many healthcare workers lack the necessary training in IPC protocols and infection management skills, and the proportion of trained personnel is low across the continent [15, 18, 26, 30–32]. Even in facilities where staff have undergone IPC training, low staffing levels and insufficient knowledge compromise healthcare safety and the quality of service delivery. This deficiency not only jeopardizes healthcare outcomes but also increases the risk of outbreaks spreading further [16, 19, 23, 27, 28, 33, 48, 49]. Adequate staffing, comprehensive training, and support for healthcare workers are essential for effective Q&I implementation and better patient outcomes. Additionally, the review highlighted deficiencies in Africa’s capacity to screen and detect infections. Laboratory facilities and testing kits were limited, unable to cope with the volume of suspected cases. Infection preparedness and Q&I implementation were further hindered by a lack of attention to staff welfare. Only two of the 23 studies reviewed discussed healthcare workers’ welfare during outbreaks, revealing inadequate working conditions such as insufficient pay, poor accommodation, and a lack of insurance or protection against infections [50]. Poor working conditions are exacerbated during outbreaks due to increased workloads and heightened risks, leading to job dissatisfaction and compromising healthcare system resilience [50–52].

Financial constraints further hampered Q&I efforts across Africa. Only two reviewed studies discussed the role of funding in implementing Q&I measures, but other literature confirms that insufficient financial resources are a major challenge for infection control in Africa. Countries struggled to allocate resources effectively during outbreaks, resulting in irregular compliance with IPC regulations and poor governance of Q&I measures [51, 52]. These challenges, coupled with infrastructural and human resource limitations, made it difficult for many African countries to effectively manage infectious disease outbreaks and implement Q&I strategies. Beyond the healthcare system, social and cultural factors also affected outbreak control in Africa. Rumors, misinformation, stigmatization, and poor communication hindered efforts to contain infections. Some patients were reluctant to comply with Q&I measures due to fears of contracting infections within isolation facilities, while healthcare workers experienced increased stress due to their workloads and the risk of infection. These challenges were similar to those reported in other countries such as Finland, China, and Canada [53–55].

The review also highlighted the negative psychological effects of quarantine. Many individuals in isolation reported experiencing boredom, poor hygiene, and low-quality meals. Some developed symptoms of anxiety, depression, stress, and insomnia, while healthcare workers also showed signs of anxiety and depression. These mental health challenges were similarly reported in other parts of the world, including Europe and Asia [54, 56, 57]. Coping mechanisms such as emotion-focused and problem-focused strategies, as well as personal and social support, were employed by both patients and healthcare workers to deal with these psychological challenges. While the review provides valuable insights into the preparedness of African healthcare systems to manage infectious disease outbreaks, it also reveals significant gaps in the literature. Only one study focused on the experiences of quarantined individuals, and only one intervention study was included. Additionally, socio-cultural, ethical, and financial aspects of Q&I were not thoroughly addressed. Further research should explore these dimensions, as well as the comparative effectiveness of Q&I practices for different disease conditions, to improve Africa’s response to future outbreaks.

## Conclusions

The reviewed studies have highlighted the significant challenges faced by Q&I measures in the healthcare system, particularly in managing the spread of infectious diseases like Zika, monkeypox, Ebola, and coronavirus in Africa. Q&I are public health measures that aim to protect the public by preventing exposure to individuals with or potentially having an infectious disease. However, low human resource capacity and inefficiencies within the healthcare system often hinder the implementation of Q&I in Africa. This review suggests that basic intensive care training should be made available to healthcare workers, more investment in health resources is needed to procure diagnostic machines, support staff, and build more infrastructure at a national level, and strengthening national mental health strategies, including local-level mental health workers, is necessary to address the psychosocial burden of Q&I. Continuous efforts to identify and address these challenges are crucial to enhancing health emergency preparedness in Africa.

## Supplementary Information

Supplementary material 1.

## Data Availability

Data sharing is not applicable to this article as no datasets were generated or analysed during the current study.
