# Supplementary material for: Challenges associated with the implementation of institutional quarantine and isolation strategies during major multicountry viral outbreaks in Africa (2000–2023): a scoping review
Source: Glob Health Res Policy. 2024 Oct 18;9:44. doi: 10.1186/s41256-024-00385-8 (PMC11489991; doi:10.1186/s41256-024-00385-8)
Supplement: Supplementary file 1 — Supplementary material 1. [file 41256_2024_385_MOESM1_ESM.docx]

**SUPPLEMENTARY FILE**

**Table S1. Search string for PubMed database search**

| **Tag** | **Subject search** | **Search String** |
| --- | --- | --- |
| #1 | Quarantine | ((((Quarantine[Title/Abstract]) OR (isolat*[Title/Abstract])) OR (separat*[Title/Abstract])) OR (seclu*[Title/Abstract])) OR (deten*[Title/Abstract]) |
| #2 | Viral infectious diseases of interest | ((((Ebola[Title/Abstract]) OR (COVID*[Title/Abstract])) OR (corona*[Title/Abstract])) OR (sars-cov-2[Title/Abstract])) OR (Lassa[Title/Abstract]) |
| #3 | Pandemic | ((pandemic[Title/Abstract]) OR (outbreak[Title/Abstract])) OR (widespread[Title/Abstract]) |
| #4 | African countries, dependencies, and territories | (((((((((((((((((((((((((((((((((((((((((((((((((((((((((((Algeria[MeSH Terms]) OR (Angola[MeSH Terms])) OR (Benin[MeSH Terms])) OR (Botswana[MeSH Terms])) OR (burkina faso[MeSH Terms])) OR (burundi[MeSH Terms])) OR (cabo verde[MeSH Terms])) OR (cape verde[MeSH Terms])) OR (cameroon[MeSH Terms])) OR (central african republic[MeSH Terms])) OR (chad[MeSH Terms])) OR (comoros[MeSH Terms])) OR (congo[MeSH Terms])) OR (ivory coast[MeSH Terms])) OR (cote d ivoire[MeSH Terms])) OR (djibouti[MeSH Terms])) OR (democratic republic of congo[MeSH Terms])) OR (egypt[MeSH Terms])) OR (equatorial guinea[MeSH Terms])) OR (eritrea[MeSH Terms])) OR (eswatini[MeSH Terms])) OR (ethiopia[MeSH Terms])) OR (gabon[MeSH Terms])) OR (gambia[MeSH Terms])) OR (ghana[MeSH Terms])) OR (guinea[MeSH Terms])) OR (guinea bissau[MeSH Terms])) OR (kenya[MeSH Terms])) OR (lesotho[MeSH Terms])) OR (liberia[MeSH Terms])) OR (libya[MeSH Terms])) OR (madagascar[MeSH Terms])) OR (malawi[MeSH Terms])) OR (mali[MeSH Terms])) OR (mauritania[MeSH Terms])) OR (mauritius[MeSH Terms])) OR (morocco[MeSH Terms])) OR (mozambique[MeSH Terms])) OR (namibia[MeSH Terms])) OR (niger[MeSH Terms])) OR (nigeria[MeSH Terms])) OR (rwanda[MeSH Terms])) OR (sao tome and principe[MeSH Terms])) OR (senegal[MeSH Terms])) OR (seychelles[MeSH Terms])) OR (sierra leone[MeSH Terms])) OR (somalia[MeSH Terms])) OR (south africa[MeSH Terms])) OR (south sudan[MeSH Terms])) OR (sudan[MeSH Terms])) OR (tanzania[MeSH Terms])) OR (togo[MeSH Terms])) OR (tunisia[MeSH Terms])) OR (uganda[MeSH Terms])) OR (zambia[MeSH Terms])) OR (zimbabwe[MeSH Terms])) OR (reunion[MeSH Terms])) OR (saint helena[MeSH Terms])) OR (western sahara[MeSH Terms])) OR (mayotte[MeSH Terms]) |
| #5 | #1 AND #2 AND #3 AND #4 | (((#1) AND (#2)) AND (#3)) AND (#4) |

**Table S2. Search string for SCOPUS database search**

| **Tag** | **Subject search** | **Search String** |
| --- | --- | --- |
| #1 | Quarantine | ( TITLE-ABS-KEY ( quarantine )  OR  TITLE-ABS-KEY ( isolat* )  OR  TITLE-ABS-KEY ( separat* )  OR  TITLE-ABS-KEY ( seclu* )  OR  TITLE-ABS-KEY ( deten* ) ) |
| #2 | Viral infectious diseases of interest | ( TITLE-ABS-KEY ( ebola )  OR  TITLE-ABS-KEY ( covid* )  OR  TITLE-ABS-KEY ( corona* )  OR  TITLE-ABS-KEY ( sars-cov-2 )  OR  TITLE-ABS-KEY ( lassa ) ) |
| #3 | Pandemic | ( TITLE-ABS-KEY ( pandemic )  OR  TITLE-ABS-KEY ( outbreak )  OR  TITLE-ABS-KEY ( widespread ) ) |
| #4 | African countries, dependencies, and territories | ( ( TITLE-ABS-KEY ( angola )  OR  TITLE-ABS-KEY ( benin )  OR  TITLE-ABS-KEY ( botswana )  OR  TITLE-ABS-KEY ( "burkina faso" )  OR  TITLE-ABS-KEY ( burundi )  OR  TITLE-ABS-KEY ( cameroon )  OR  TITLE-ABS-KEY ( "cabo verde" )  OR  TITLE-ABS-KEY ( "cape verde" )  OR  TITLE-ABS-KEY ( "central african republic" )  OR  TITLE-ABS-KEY ( chad )  OR  TITLE-ABS-KEY ( comoros )  OR  TITLE-ABS-KEY ( congo )  OR  TITLE-ABS-KEY ( "ivory coast" )  OR  TITLE-ABS-KEY ( "democratic republic of congo" )  OR  TITLE-ABS-KEY ( djibouti )  OR  TITLE-ABS-KEY ( "equatorial guinea" )  OR  TITLE-ABS-KEY ( eritrea )  OR  TITLE-ABS-KEY ( ethiopia )  OR  TITLE-ABS-KEY ( gabon )  OR  TITLE-ABS-KEY ( gambia )  OR  TITLE-ABS-KEY ( ghana )  OR  TITLE-ABS-KEY ( guinea )  OR  TITLE-ABS-KEY ( guinea-bissau )  OR  TITLE-ABS-KEY ( kenya )  OR  TITLE-ABS-KEY ( lesotho )  OR  TITLE-ABS-KEY ( liberia )  OR  TITLE-ABS-KEY ( madagascar )  OR  TITLE-ABS-KEY ( malawi )  OR  TITLE-ABS-KEY ( mali )  OR  TITLE-ABS-KEY ( mauritania )  OR  TITLE-ABS-KEY ( mauritius )  OR  TITLE-ABS-KEY ( mayotte )  OR  TITLE-ABS-KEY ( mozambique )  OR  TITLE-ABS-KEY ( namibia )  OR  TITLE-ABS-KEY ( niger )  OR  TITLE-ABS-KEY ( nigeria )  OR  TITLE-ABS-KEY ( reunion )  OR  TITLE-ABS-KEY ( rwanda )  OR  TITLE-ABS-KEY ( "saint helena" )  OR  TITLE-ABS-KEY ( sao  AND tome  AND  principe )  OR  TITLE-ABS-KEY ( senegal )  OR  TITLE-ABS-KEY ( seychelles )  OR  TITLE-ABS-KEY ( "sierra leone" )  OR  TITLE-ABS-KEY ( somalia )  OR  TITLE-ABS-KEY ( "south africa" )  OR  TITLE-ABS-KEY ( "south sudan" ) ) )  OR  ( ( TITLE-ABS-KEY ( eswatini )  OR  TITLE-ABS-KEY ( togo )  OR  TITLE-ABS-KEY ( uganda )  OR  TITLE-ABS-KEY ( zambia )  OR  TITLE-ABS-KEY ( zimbabwe )  OR  TITLE-ABS-KEY ( egypt )  OR  TITLE-ABS-KEY ( libya )  OR  TITLE-ABS-KEY ( algeria )  OR  TITLE-ABS-KEY ( tunisia )  OR  TITLE-ABS-KEY ( morocco )  OR  TITLE-ABS-KEY ( "western sahara" )  OR  TITLE-ABS-KEY ( sudan )  OR  TITLE-ABS-KEY ( tunisia ) ) ) |
| #5 | #1 AND #2 AND #3 AND #4 | (#1) AND (#2) AND (#3) AND (#4) |

**Table S3. Search string for other database (AMED – The Allied and Complementary Medicine Database; CINAHL Complete; Dentistry and Oral Sciences Source; SPORTDiscus with Full Text; APA PsycArticles; Psychology and Behavioral Sciences Collection; APA PsycInfo and CINAHL Ultimate) search via EBSCO interface**

| **Tag** | **Subject search** | **Search String** |
| --- | --- | --- |
| S1 | Quarantine | AB Quarantine OR AB isolat* OR AB separat* OR AB seclu* OR AB deten* |
| S2 | Viral infectious diseases of interest | AB Ebola OR AB COVID* OR AB corona* OR AB sars-cov-2 OR AB Lassa |
| S3 | Pandemic | AB pandemic OR AB outbreak OR AB widespread |
| S4 | African countries, dependencies, and territories | AB algeria OR AB angola OR AB benin OR AB botswana OR AB burkina faso OR AB burundi OR AB cape verde OR AB cabo verde OR AB cameroon OR AB central african republic OR AB chad OR AB comoros OR AB congo OR AB cote d'ivoire OR AB ivory coast OR AB djibouti OR AB democratic republic of congo OR AB egypt OR AB equatorial guinea OR AB eritrea OR AB eswatini OR AB ethiopia OR AB gabon OR AB gambia OR AB ghana OR AB guinea OR AB guinea bissau OR AB kenya OR AB lesotho OR AB liberia OR AB libya OR AB madagascar OR AB malawi OR AB mali OR AB mauritania OR AB mauritius OR AB morocco OR AB mozambique OR AB namibia OR AB niger OR AB nigeria OR AB rwanda OR AB ( sao tome and principe ) OR AB senegal OR AB seychelles OR AB sierra leone OR AB somalia OR AB south Africa OR AB south sudan OR AB sudan OR AB tanzania OR AB togo OR AB tunisia OR AB uganda OR AB zambia OR AB zimbabwe OR AB reunion OR AB saint helena OR AB western sahara OR AB mayotte |
| S5 | S1 AND S2 AND S3 AND S4 | S1 AND S2 AND S3 AND S4 |

**TABLE S4. List of articles whose full texts were screened for inclusion/exclusion into the scoping review.**

| **No.** | **CITATION** | **INCLUDED** | **EXCLUDED (REASONS)** |
| --- | --- | --- | --- |
| 1 | Ndejjo, R., Naggayi, G., Tibiita, R., Mugahi, R., & Kibira, S. P. S. (2021). Experiences of persons in COVID-19 institutional quarantine in Uganda: a qualitative study. *BMC public health*, *21*(1), 482. https://doi.org/10.1186/s12889-021-10519-z | Yes |  |
| 2 | Ogoina, D., Mahmood, D., Oyeyemi, A. S., Okoye, O. C., Kwaghe, V., Habib, Z., Unigwe, U., Iroezindu, M. O., Garbati, M. A., Rotifa, S., Adekanmbi, O., Garba, I., Dayyab, F. M., Ibrahim, S. M., Kida, I. M., Adamu, A., Alasia, D., Awang, S. K., Ohaju-Obodo, J. O., Usman, R., … Habib, A. G. (2021). A national survey of hospital readiness during the COVID-19 pandemic in Nigeria. *PloS one*, *16*(9), e0257567. https://doi.org/10.1371/journal.pone.0257567 | Yes |  |
| 3 | Abd El-Raheem, G. O. H., Yousif, M. A. A., Mohamed, D. S. I., Farah, R. O. G., Bukhari, M. E. A., Mohamed, N. F. E., Ahmad, M. O., & Saeed, B. K. I. (2022). Extent of COVID-19 Healthcare Services of Isolation Center of Private Hospital across Khartoum State, Sudan. *Journal of environmental and public health*, *2022*, 6745813. https://doi.org/10.1155/2022/6745813 |  | Yes (Wrong study objectives) |
| 4 | Arthur, R. F., Horng, L. M., Bolay, F. K., Tandanpolie, A., Gilstad, J. R., Tantum, L. K., & Luby, S. P. (2022). Community trust of government and non-governmental organizations during the 2014-16 Ebola epidemic in Liberia. *PLoS neglected tropical diseases*, *16*(1), e0010083. https://doi.org/10.1371/journal.pntd.0010083 |  | Yes (Wrong study objectives) |
| 5 | Bassey, E. B., Kazadi Mulomb, W., Ahmed Khedr, A. M., Mpazanje, R. G., Onyibe, R. I., Kolude, O. O., Marcus, O., Alawale, O., Ogunlaja, O., Oluwatobi, A. I., Adedamola, A. T., Olayiwola, S. O., & Ladipo, T. O. (2022). COVID-19 hot-spot strategy: a special innovation in pandemic response, Oyo State Nigeria. *BMC public health*, *22*(1), 233. https://doi.org/10.1186/s12889-022-12675-2 |  | Yes (Wrong study objectives) |
| 6 | David, N., & Mash, R. (2020). Community-based screening and testing for Coronavirus in Cape Town, South Africa: Short report. *African journal of primary health care & family medicine*, *12*(1), e1–e3. https://doi.org/10.4102/phcfm.v12i1.2499 |  | Yes (Wrong publication type) |
| 7 | Adokiya, M. N., & Awoonor-Williams, J. K. (2016). Ebola virus disease surveillance and response preparedness in northern Ghana. *Global health action*, *9*, 29763. https://doi.org/10.3402/gha.v9.29763 | Yes |  |
| 8 | Pellecchia, U., Crestani, R., Decroo, T., Van den Bergh, R., & Al-Kourdi, Y. (2015). Social Consequences of Ebola Containment Measures in Liberia. *PloS one*, *10*(12), e0143036. https://doi.org/10.1371/journal.pone.0143036 | Yes |  |
| 9 | Kharroubi, G., Cherif, I., Amor, S. H., Zribi, M., Atigue, W. B., Ouali, U., & Bettaieb, J. (2021). Mental health status of adults under institutional quarantine: a cross-sectional survey in Tunisia. *The Pan African medical journal*, *40*, 197. https://doi.org/10.11604/pamj.2021.40.197.31112 | Yes |  |
| 10 | Fawole, O. I., Bello, S., Adebowale, A. S., Bamgboye, E. A., Salawu, M. M., Afolabi, R. F., Dairo, M. D., Namale, A., Kiwanuka, S., Monje, F., Namuhani, N., Kabwama, S., Kizito, S., Ndejjo, R., Seck, I., Diallo, I., Makhtar, M., Leye, M., Ndiaye, Y., Fall, M., … Wanyenze, R. (2023). COVID-19 surveillance in Democratic Republic of Congo, Nigeria, Senegal and Uganda: strengths, weaknesses and key Lessons. *BMC public health*, *23*(1), 835. https://doi.org/10.1186/s12889-023-15708-6 | Yes |  |
| 11 | Nyenswah, T., Blackley, D. J., Freeman, T., Lindblade, K. A., Arzoaquoi, S. K., Mott, J. A., Williams, J. N., Halldin, C. N., Kollie, F., Laney, A. S., & Centers for Disease Control and Prevention (CDC) (2015). Community quarantine to interrupt Ebola virus transmission - Mawah Village, Bong County, Liberia, August-October, 2014. *MMWR. Morbidity and mortality weekly report*, *64*(7), 179–182. |  | Yes (Wrong publication type) |
| 12 | Olani, A. B., Degefa, N., Aschalew, Z., Kassa, M., Feleke, T., Gura, G., & Wambete, S. N. (2022). Exploring experiences of quarantined people during the early phase of COVID-19 outbreak in Southern Nations Nationalities and Peoples' Region of Ethiopia: A qualitative study. *PloS one*, *17*(9), e0275248. https://doi.org/10.1371/journal.pone.0275248 | Yes |  |
| 13 | Nikolaeva, A., & Versnel, J. (2022). Analytical observational study evaluating global pandemic preparedness and the effectiveness of early COVID-19 responses in Ethiopia, Nigeria, Singapore, South Korea, Sweden, Taiwan, UK and USA. *BMJ open*, *12*(2), e053374. https://doi.org/10.1136/bmjopen-2021-053374 |  | Yes (Wrong study objectives) |
| 14 | Ndoungué, V. F., Ngapagna, A. N., Kouadio, S. A., Djinguebey, R., Gnigninanjouena, O., Eyangoh, S., Nguefack-Tsague, G., Djeunga, H. C. N., & Njajou, O. (2022). Assessing core capacities for addressing public health emergencies of international concern at designated points of entry in cameroon during the COVID-19 Pandemic. *BMC public health*, *22*(1), 2197. https://doi.org/10.1186/s12889-022-14614-7 | Yes |  |
| 15 | Kpanake, L., Gossou, K., Sorum, P. C., & Mullet, E. (2016). Misconceptions about Ebola virus disease among lay people in Guinea: Lessons for community education. *Journal of public health policy*, *37*(2), 160–172. https://doi.org/10.1057/jphp.2016.1 |  | Yes (Wrong study objectives) |
| 16 | Takyiakwaa, D., Tuoyire, D. A., Abraham, S. A., Agyare, E. A., Amoah, J. O., Owusu-Sarpong, A. A., Omona, K., Obiri-Yeboah, D., & Doku, D. T. (2023). Culture and pandemic control at cross-roads: navigating the burial guidelines for COVID-19-related deaths in a Ghanaian setting. *BMC health services research*, *23*(1), 519. https://doi.org/10.1186/s12913-023-09421-8 |  | Yes (Wrong study outcomes) |
| 17 | Youssef, N., Mostafa, A., Ezzat, R., Yosef, M., & El Kassas, M. (2020). Mental health status of health-care professionals working in quarantine and non-quarantine Egyptian hospitals during the COVID-19 pandemic. *Eastern Mediterranean health journal = La revue de sante de la Mediterranee orientale = al-Majallah al-sihhiyah li-sharq al-mutawassit*, *26*(10), 1155–1164. https://doi.org/10.26719/emhj.20.116 |  | Yes (Wrong study outcomes) |
| 18 | Olu, O., Kargbo, B., Kamara, S., Wurie, A. H., Amone, J., Ganda, L., Ntsama, B., Poy, A., Kuti-George, F., Engedashet, E., Worku, N., Cormican, M., Okot, C., Yoti, Z., Kamara, K. B., Chitala, K., Chimbaru, A., & Kasolo, F. (2015). Epidemiology of Ebola virus disease transmission among health care workers in Sierra Leone, May to December 2014: a retrospective descriptive study. *BMC infectious diseases*, *15*, 416. https://doi.org/10.1186/s12879-015-1166-7 |  | Yes (Wrong study outcomes) |
| 19 | Habtamu, K., Desie, Y., Asnake, M., Lera, E. G., & Mequanint, T. (2021). Psychological distress among Ethiopian migrant returnees who were in quarantine in the context of COVID-19: institution-based cross-sectional study. *BMC psychiatry*, *21*(1), 424. https://doi.org/10.1186/s12888-021-03429-2 | Yes |  |
| 20 | Adebimpe, W. O., & Ibirongbe, D. O. (2019). Exploring the Knowledge and Preventive Practices on Isolation Precaution and Quarantine Among Health Care Workers in Ondo State, Nigeria. *Annals of global health*, *85*(1), 72. https://doi.org/10.5334/aogh.2454 | Yes |  |
| 21 | Sanni, U. A., Offiong, U. M., Anigilaje, E. A., Airede, K. I., & Imam, A. (2021). A pre-COVID-19 assessment of aspects of the school health programme in some selected Nigerian primary schools: implications for school re-opening during the COVID-19 pandemic in developing country contexts. *BMC public health*, *21*(1), 1214. https://doi.org/10.1186/s12889-021-11258-x | Yes |  |
| 22 | Abramowitz, S. A., McLean, K. E., McKune, S. L., Bardosh, K. L., Fallah, M., Monger, J., Tehoungue, K., & Omidian, P. A. (2015). Community-centered responses to Ebola in urban Liberia: the view from below. *PLoS neglected tropical diseases*, *9*(4), e0003706. https://doi.org/10.1371/journal.pntd.0003706 |  | Yes (Wrong study objectives) |
| 23 | Kayiga, H., Genevive, D. A., Amuge, P. M., Ssemata, A. S., Nanzira, R. S., & Nakimuli, A. (2021). Lived experiences of frontline healthcare providers offering maternal and newborn services amidst the novel corona virus disease 19 pandemic in Uganda: A qualitative study. *PloS one*, *16*(12), e0259835. https://doi.org/10.1371/journal.pone.0259835 |  | Yes (Wrong study objectives) |
| 24 | Oji, M. O., Haile, M., Baller, A., Tremblay, N., Mahmoud, N., Gasasira, A., Ladele, V., Cooper, C., Kateh, F. N., Nyenswah, T., & Nsubuga, P. (2018). Implementing infection prevention and control capacity building strategies within the context of Ebola outbreak in a "Hard-to-Reach" area of Liberia. *The Pan African medical journal*, *31*, 107. https://doi.org/10.11604/pamj.2018.31.107.15517 | Yes |  |
| 25 | Pedi, D., Gillespie, A., Bedson, J., Jalloh, M. F., Jalloh, M. B., Kamara, A., Bertram, K., Owen, K., Jalloh, M. A., & Conte, L. (2017). The Development of Standard Operating Procedures for Social Mobilization and Community Engagement in Sierra Leone During the West Africa Ebola Outbreak of 2014-2015. *Journal of health communication*, *22*(sup1), 39–50. https://doi.org/10.1080/10810730.2016.1212130 | Yes |  |
| 26 | Stehling-Ariza, T., Rosewell, A., Moiba, S. A., Yorpie, B. B., Ndomaina, K. D., Jimissa, K. S., Leidman, E., Rijken, D. J., Basler, C., Wood, J., & Manso, D. (2016). The impact of active surveillance and health education on an Ebola virus disease cluster - Kono District, Sierra Leone, 2014-2015. *BMC infectious diseases*, *16*(1), 611. https://doi.org/10.1186/s12879-016-1941-0 |  | Yes (Wrong study outcomes) |
| 27 | Makinde, O. A., & Odimegwu, C. O. (2018). A qualitative inquiry on the status and adequacy of legal instruments establishing infectious disease surveillance in Nigeria. *The Pan African medical journal*, *31*, 22. https://doi.org/10.11604/pamj.2018.31.22.14119 |  | Yes (Wrong study outcomes) |
| 28 | GebreEyesus, F. A., Geleta, O. T., Shiferaw, B. Z., Tarekegn, T. T., Amlak, B. T., Emeria, M. S., Terefe, T. F., Temere, B. C., Mewahegn, A. A., Jimma, M. S., Chanie, E. S., Misganaw, N. M., Degu, F. S., & Eshetu, M. A. (2023). Health care providers' preparedness and health care protection against the third wave of COVID-19 pandemics in a resource-limited setting in Southwest Ethiopia: a multi-center cross-sectional study. *The Pan African medical journal*, *44*, 53. https://doi.org/10.11604/pamj.2023.44.53.31428 | Yes |  |
| 29 | Wolfe, C. M., Hamblion, E. L., Schulte, J., Williams, P., Koryon, A., Enders, J., Sanor, V., Wapoe, Y., Kwayon, D., Blackley, D. J., Laney, A. S., Weston, E. J., Dokubo, E. K., Davies-Wayne, G., Wendland, A., Daw, V. T. S., Badini, M., Clement, P., Mahmoud, N., Williams, D., … Fallah, M. (2017). Ebola virus disease contact tracing activities, lessons learned and best practices during the Duport Road outbreak in Monrovia, Liberia, November 2015. *PLoS neglected tropical diseases*, *11*(6), e0005597. https://doi.org/10.1371/journal.pntd.0005597 |  | Yes (Wrong study outcomes) |
| 30 | Awoonor-Williams, J. K., Moyer, C. A., & Adokiya, M. N. (2021). Self-reported challenges to border screening of travelers for Ebola by district health workers in northern Ghana: An observational study. *PloS one*, *16*(1), e0245039. https://doi.org/10.1371/journal.pone.0245039 | Yes |  |
| 31 | Kwaghe, A. V., Ilesanmi, O. S., Amede, P. O., Okediran, J. O., Utulu, R., & Balogun, M. S. (2021). Stigmatization, psychological and emotional trauma among frontline health care workers treated for COVID-19 in Lagos State, Nigeria: a qualitative study. *BMC health services research*, *21*(1), 855. https://doi.org/10.1186/s12913-021-06835-0 |  | Yes (Wrong study outcomes) |
| 32 | Ijarotimi, I. T., Ilesanmi, O. S., Aderinwale, A., Abiodun-Adewusi, O., & Okon, I. M. (2018). Knowledge of Lassa fever and use of infection prevention and control facilities among health care workers during Lassa fever outbreak in Ondo State, Nigeria. *The Pan African medical journal*, *30*, 56. https://doi.org/10.11604/pamj.2018.30.56.13125 | Yes |  |
| 33 | Asare, I. T., Douglas, M., Kye-Duodu, G., & Manu, E. (2023). Challenges and opportunities for improved contact tracing in Ghana: experiences from Coronavirus disease-2019-related contact tracing in the Bono region. *BMC infectious diseases*, *23*(1), 335. https://doi.org/10.1186/s12879-023-08317-6 | Yes |  |
| 34 | Joy Okwor, T., Gatua, J., Umeokonkwo, C. D., Abah, S., Ike, I. F., Ogunniyi, A., Ipadeola, O., Attah, T., Assad, H., Dooga, J., Olayinka, A., Abubakar, J., Oladejo, J., Aderinola, O., Eneh, C., Ilori, E., Ibekwe, P., Ochu, C., & Ihekweazu, C. (2022). An assessment of infection prevention and control preparedness of healthcare facilities in Nigeria in the early phase of the COVID-19 pandemic (February-May 2020). *Journal of infection prevention*, *23*(3), 101–107. https://doi.org/10.1177/17571774211060418 | Yes |  |
| 35 | Obionu, I. M., Ochu, C. L., Ukponu, W., Okwor, T., Dan-Nwafor, C., Ilori, E., & Ihekweazu, C. (2021). Evaluation of infection prevention and control practices in Lassa fever treatment centers in north-central Nigeria during an ongoing Lassa fever outbreak. *Journal of infection prevention*, *22*(6), 275–282. https://doi.org/10.1177/17571774211035838 | Yes |  |
| 36 | Cherif, I., Kharroubi, G., Haj Amor, S., Zribi, M., Ouali, U., & Bettaieb, J. (2021). Impact of mandatory institutional quarantine on sleep quality: A cross sectional Tunisian study. *European Journal of Public Health*, *31*(Supplement_3), ckab165-040. |  | Yes (Wrong publication type) |
| 37 | Cherif, I., Kharroubi, G., Haj Amor, S., Zribi, M., Ouali, U., & Bettaieb, J. (2021). Anxiety and depression among individuals in mandatory institutional quarantine in Tunisia. *European Journal of Public Health*, *31*(Supplement_3), ckab165-042. <https://doi.org/10.1093/eurpub/ckab165.042> |  | Yes (Wrong publication type) |
| 38 | Misgana, T., Tesfaye, D., Tariku, M., Ali, T., Alemu, D., & Dessie, Y. (2022). Psychological Burden and Associated Factors of the COVID-19 Pandemic on People in Quarantine and Isolation Centers in Ethiopia: A Cross-Sectional Study. *Frontiers in psychiatry*, *12*, 753383. https://doi.org/10.3389/fpsyt.2021.753383 | Yes |  |
| 39 | Desie, Y., Habtamu, K., Asnake, M., Gina, E., & Mequanint, T. (2021). Coping strategies among Ethiopian migrant returnees who were in quarantine in the time of COVID-19: a center-based cross-sectional study. *BMC psychology*, *9*(1), 192. https://doi.org/10.1186/s40359-021-00699-z | Yes |  |
| 40 | Blackley, D. J., Lindblade, K. A., Kateh, F., Broyles, L. N., Westercamp, M., Neatherlin, J. C., Pillai, S. K., Tucker, A., Mott, J. A., Walke, H., Nyenswah, T., & Centers for Disease Control and Prevention (CDC) (2015). Rapid intervention to reduce Ebola transmission in a remote village - Gbarpolu County, Liberia, 2014. *MMWR. Morbidity and mortality weekly report*, *64*(7), 175–178. |  | Yes (Wrong publication type) |
| 41 | Lamontagne, F., Fowler, R. A., Adhikari, N. K., Murthy, S., Brett-Major, D. M., Jacobs, M., Uyeki, T. M., Vallenas, C., Norris, S. L., Fischer, W. A., 2nd, Fletcher, T. E., Levine, A. C., Reed, P., Bausch, D. G., Gove, S., Hall, A., Shepherd, S., Siemieniuk, R. A., Lamah, M. C., Kamara, R., … Guyatt, G. H. (2018). Evidence-based guidelines for supportive care of patients with Ebola virus disease. *Lancet (London, England)*, *391*(10121), 700–708. https://doi.org/10.1016/S0140-6736(17)31795-6 |  | Yes (Wrong publication type) |
| 42 | Bettini, A., Lapa, D., & Garbuglia, A. R. (2023). Diagnostics of Ebola virus. *Frontiers in public health*, *11*, 1123024. https://doi.org/10.3389/fpubh.2023.1123024 |  | Yes (Wrong publication type) |
| 43 | Aidonojie, P. A., Okuonghae, N., & Ukhurebor, K. E. (2022). The Legal Rights and Challenges of COVID-19 Patients Accessing Private Healthcare in Nigeria. *BESTUUR*, *10*(2), 183-197. https://doi.org/10.20961/bestuur.v10i2.68118. | Yes |  |
| 44 | Idrees, M. H. D., & Bashir, M. M. I. (2023). The psychological impact of the COVID-19 pandemic on the Sudanese healthcare workers in quarantine centers: a cross-sectional study 2020–2021. *Middle East Current Psychiatry*, *30*(1), 1-8. https://doi.org/10.1186/s43045-023-00281-w | Yes |  |
| 45 | Delamou, A., Sow, A., Fofana, T. O., Sidibé, S., Kourouma, K., Sandouno, M., Touré, A., Tounkara, T. M., Le Marcis, F., & Van Damme, W. (2022). A rapid assessment of health system preparedness and response to the COVID-19 pandemic in Guinea. *Journal of public health in Africa*, *13*(2), 1475. https://doi.org/10.4081/jphia.2022.1475 | Yes |  |
| 46 | Kruger, P., & Karim, S. A. (2022). A human rights-based approach to coercive public health interventions: lessons from the HIV and COVID-19 pandemics. *African journal of AIDS research : AJAR*, *21*(2), 123–131. https://doi.org/10.2989/16085906.2022.2073897 |  | Yes (Wrong publication type) |
